# Complete Resolution of Metastatic Gallbladder Cancer after Standard Gemcitabine-Cisplatin Combination Therapy

**DOI:** 10.7759/cureus.415

**Published:** 2015-12-17

**Authors:** Young Soo Rho, Ivan Barrera, Peter Metrakos, Petr Kavan

**Affiliations:** 1 Department of Oncology, McGill University Health Centre, Montreal, QC, Canada; 2 Department of Medical Oncology, Sir Mortimer B. Davis Jewish General Hospital, Segal Cancer Centre, McGill University, Montreal, QC, Canada; 3 Section of Hepatobiliary and Transplant Surgery, McGill University Health Centre, Montreal, QC, Canada; 4 Departments of Oncology and Medical Oncology, Sir Mortimer B. Davis Jewish General Hospital, Segal Cancer Centre, McGill University, Montreal, QC, Canada

**Keywords:** gallbladder cancer, biliary tract cancer, gemcitabine, cisplatin, complete response, gastrointestinal malignancy

## Abstract

Gallbladder carcinoma (GBC) is a rare and deadly disease. The only curative option is a total surgical resection. If the disease is inoperable, palliative combination chemotherapy with gemcitabine-cisplatin remains the standard of care. We present here a case of a 47-year-old gentleman diagnosed with metastatic GBC who saw a complete resolution of his disease with seven cycles of standard gemcitabine-cisplatin chemotherapy. This case illustrates the importance of multidisciplinary care to explore all available options to provide optimal and tailored patient care.

## Introduction

Gallbladder carcinoma (GBC) is an uncommon and poorly understood disease with only 500 reported cases in Canada in 2010 [1]. The aggressive nature of GBC makes it extremely difficult to control. In the management of inoperable GBCs, i.e. locally advanced and metastatic disease, combined gemcitabine-cisplatin (gem/cis) chemotherapy remains to be the standard of care. Unfortunately, the overall survival (OS) in the aforementioned setting remains less than 12 months [2]. Often in clinical practice, due to this poor prognosis, patients tend to forego any further treatment. However, we present here a case of an extraordinary response leading to clinical resolution of metastatic GBC using standard gem/cis chemotherapy.

## Case presentation

**Figure 1:**
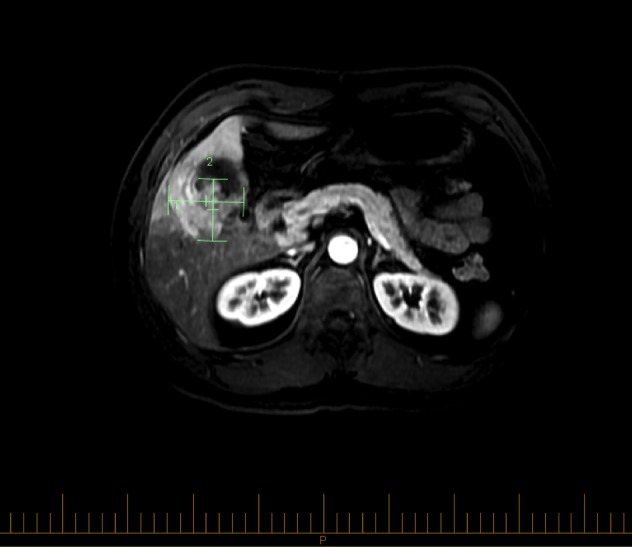
MRI with gadolinium prior to initial surgical exploration A posterolateral gallbladder mass extending to the abdominal wall muscle and liver.

In 2013, a 47-year-old Native American man presented to his family doctor complaining of a three-month history of epigastric and right hypochondrial pain. His background medical history was only significant for obstructive sleep apnea. He denied any hepato-pancreato-biliary diseases. He was not taking any regular medications. He had no family history of cancer and was working as a firefighter. He drank 1-2 units of alcohol per day and was a former smoker of 20 pack years. The patient underwent an abdominal ultrasound (US) at his local hospital, which found a mass in his gallbladder. He was subsequently transferred to our tertiary cancer center to undergo further investigation and potential treatment planning. The patient’s baseline physical examination was unremarkable and his laboratory investigation only showed a mild microcytic anemia with normal hepatic function panel. Tumor markers, carcinoembryonic antigen (CEA) and CA 19-9, were also within normal ranges. Further staging by magnetic resonance imaging (MRI) was performed, which showed a nodular and contrast-enhancing mass of 47 mm x 58 mm x 72 mm, involving the posterolateral gallbladder and extending to the abdominal wall muscle and liver segments 4B and 6 (Figure 1).

**Figure 2:**
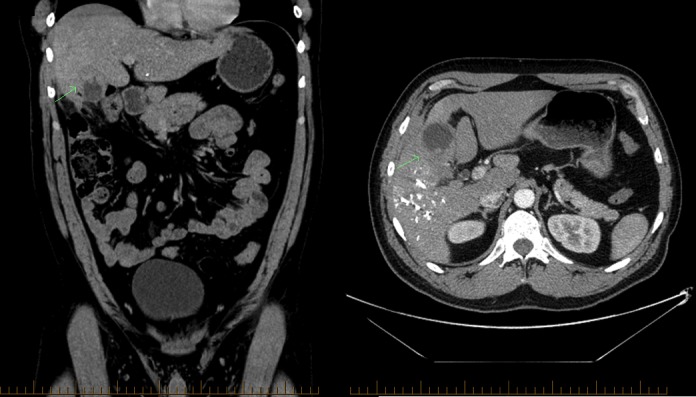
Initial staging CT of the abdomen and pelvis with contrast Coronal (left) and transverse (right) view showing extensive gallbladder mass prior to starting chemotherapy.

This was all suggestive of a malignant process. An ultrasound-guided biopsy was performed to pathologically identify the lesion; however, the resulting analysis was equivocal. The patient underwent a full workup with a computed tomography (CT) scan of the chest and upper and lower endoscopy, which did not reveal other abnormalities. After a multidisciplinary discussion, the patient was scheduled for a right trisegmentectomy with gallbladder resection. In preparation, the patient underwent a percutaneous embolization for right portal venous system without any complications. Upon exploration in the operating theatre, both hilum and hepatic artery lymph nodes were found to be positive for metastatic deposits; an intraoperative frozen section demonstrated extensive infiltrate by moderately differentiated adenocarcinoma consistent with gallbladder primary neoplasm. At the time of the surgery, no peritoneal metastases were found. Complete tumor resection was aborted. The medical oncology team saw the patient one month postoperatively for palliative treatment planning. His physical examination was unremarkable with the exception of a well healing abdominal scar and mild hepatomegaly. His Eastern Cooperative Oncology Group (ECOG) performance status was 0. His staging thorax, abdomen, and pelvis (TAP) CT scan revealed an intraluminal gallbladder mass with invasion into the peritoneum, right transversus abdominis muscle, and liver segments 4B and 5 (Figure 2). Unlike the initial MRI, several porta hepatis lymph nodes were now enlarged. No other disease was seen.

**Figure 3:**
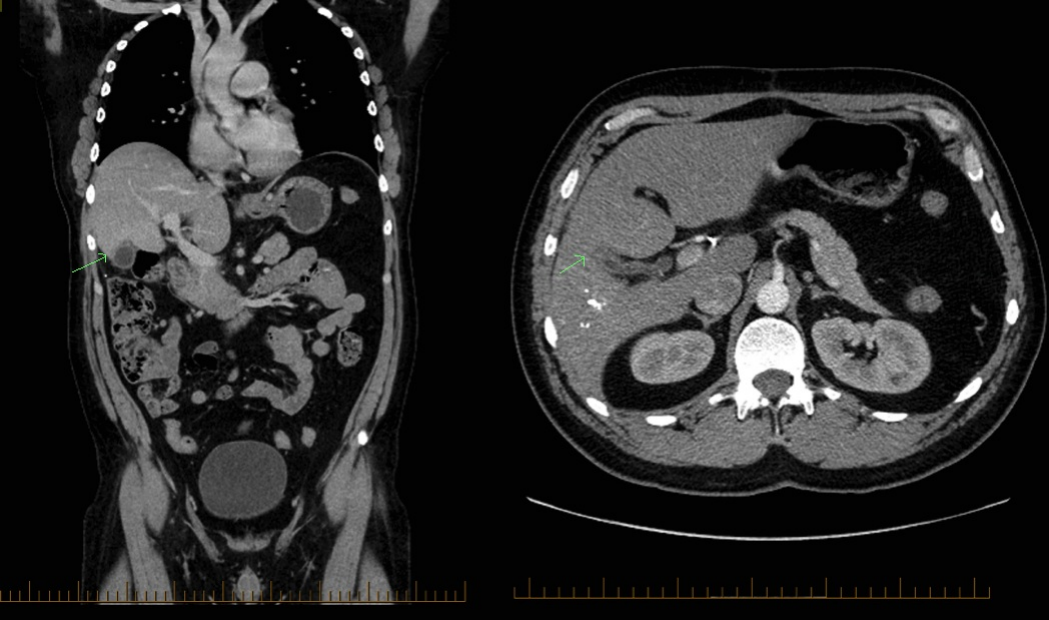
Follow-up CT of the abdomen and pelvis with contrast Coronal (left) and transverse (right) view of the CT of the abdomen and pelvis with contrast showing no sizable gallbladder mass halfway through the fourth cycle of gem/cis chemotherapy.

The patient was started on a combined chemotherapy regimen of gemcitabine (1,000 mg/m^2^) and cisplatin (25 mg/m^2^) on days 1 and 8. Halfway through the fourth cycle, the patient was re-staged with a TAP CT scan, which demonstrated significant interval improvement (Figure 3).

At that time, no sizable masses or disease was seen in the peritoneum, muscle, lymph nodes, or liver. With these findings, the patient continued another three cycles of chemotherapy, completing seven total cycles. Overall, he tolerated the treatment well with only Grade I mucositis.

The patient's six-month post-treatment TAP CT scan continued to demonstrate no radiological evidence of local recurrence or metastatic disease. In this scan, minimal thickening at the fundus and minimal fat stranding in the midline of the anterior abdominal wall were seen. The patient’s case was again discussed at a multidisciplinary meeting where it was felt that due to the radiological findings, surgical exploration and right trisegmentectomy with gallbladder resection was appropriate. The patient underwent the operation without complication and was discharged on postoperative day 7. Pathology showed no evidence of adenocarcinoma in the liver or the gallbladder; mild atypia consistent with low-grade intraepithelial neoplasia was seen in the gallbladder. One week post-surgery, the patient was readmitted for right-sided empyema, requiring admission to the intensive care unit. Fortunately, this was managed through drainage with adjunctive use of intrapleural fibrinolytic therapy alteplase and the patient was discharged, after one month. The patient has been followed regularly and continues to have no evidence of disease two years post-diagnosis.

## Discussion

Due to the rarity of the GBCs, they are often grouped as an entity in biliary tract cancers (BTCs). As such, much of the initial treatment information has been derived from other gastrointestinal malignancies; specifically, evidence of the efficacious use of gem/cis in BTC was reported in a small observational retrospective study [3]. This led to a series of randomized trials (RTs) evaluating gem/cis combined therapy in locally advanced and metastatic BTCs. The positive Phase 2 ABC-01 [4] and BT22 RTs studies [5], together with the positive Phase 3 ABC-02 RT study [2], resulted in gem/cis becoming the standard of care. Reported tumor response rate (complete response rate + partial response rate + stable disease) in the ABC-02 trial was 81.4%. However, complete response was only seen in one patient (out of 200) that received gem/cis chemotherapy. This highlights the unique case of our patient, an extraordinary responder.

## Conclusions

A case such as this highlights a number of factors in the management of patients with GBC. Despite its statistically poor prognosis, a multidisciplinary approach must be undertaken and all available options should be explored in discussion with the patient. Particular attention should be paid to patients with good responses to chemotherapy that may open opportunities for surgical intervention. Future studies should explore the mechanism of understanding chemoresistance and pursue further biomarker studies. Likewise, development of novel agents in GBCs/BTCs, including those biological/targeted therapies, are eagerly awaited.
